# Herpesviruses and SARS-CoV-2: Viral Association with Oral Inflammatory Diseases

**DOI:** 10.3390/pathogens13010058

**Published:** 2024-01-07

**Authors:** Jonathan M. Banks, Kristelle J. Capistrano, Daniela A. Brandini, Filza Zaidi, Pari Thakkar, Rani Rahat, Joel Schwartz, Afsar R. Naqvi

**Affiliations:** 1 Department of Periodontics, College of Dentistry, University of Illinois Chicago, Chicago, IL 60612, USA; jbanks25@uic.edu (J.M.B.); kcapis2@uic.edu (K.J.C.); filzahaiderzaidi@gmail.com (F.Z.); parithakkar2022@u.northwestern.edu (P.T.); rrahat3@uic.edu (R.R.); 2 Department of Diagnosis and Surgery, School of Dentistry, São Paulo State University (UNESP), Araçatuba 16015-050, SP, Brazil; daniela.brandini@unesp.br; 3 Department of Oral Medicine and Diagnostics, College of Dentistry, University of Illinois Chicago, Chicago, IL 60612, USA; joschwar@uic.edu; 4 Department of Microbiology and Immunology, College of Medicine, University of Illinois Chicago, Chicago, IL 60612, USA

**Keywords:** herpesvirus, SARS-CoV-2, oral inflammation, periodontitis, gingivitis, peri-implantitis, viral diagnostics, endodontic disease

## Abstract

The oral cavity is a niche for diverse microbes, including viruses. Members of the Herpesviridae family, comprised of dsDNA viruses, as well as severe acute respiratory syndrome coronavirus 2 (SARS-CoV-2), an ssRNA virus, are among the most prevalent viruses infecting the oral cavity, and they exhibit clinical manifestations unique to oral tissues. Viral infection of oral mucosal epithelia triggers an immune response that results in prolonged inflammation. The clinical and systemic disease manifestations of HHV have been researched extensively, and several recent studies have illuminated the relationship between HHV and oral inflammatory diseases. Burgeoning evidence suggests the oral manifestation of SARS-CoV-2 infection includes xerostomia, dysgeusia, periodontal disease, mucositis, and opportunistic viral and bacterial infections, collectively described as oral post-acute sequelae of COVID-19 (PASC). These diverse sequelae could be a result of intensified immune responses initially due to the copious production of proinflammatory cytokines: the so-called “cytokine storm syndrome”, facilitating widespread oral and non-oral tissue damage. This review explores the interplay between HHV, SARS-CoV-2, and oral inflammatory diseases such as periodontitis, endodontic disease, and peri-implantitis. Additionally, the review discusses proper diagnostic techniques for identifying viral infection and how viral diagnostics can lead to improved overall patient health.

## 1. Introduction

The human oral cavity is a niche for diverse microbial flora, including bacteria, fungi, and viruses [1,2]. Oral tissues are constantly exposed to these microbes, which form a complex ecological community that influences oral and systemic health [3]. The microbiological aspect of oral disease traditionally focuses on bacteria and fungi, but viruses are attracting increasing attention as oral pathogens. Several viruses have been discovered in the oral cavity and implicated in oral ulcers, tumors, and infectious diseases [4]. Prevalent examples of such viruses include human herpesviruses (HHV) and, more recently, severe acute respiratory syndrome coronavirus-2 (SARS-CoV-2)—the virus responsible for coronavirus disease 2019 (COVID-19).

The Herpesviridae family comprises over 100 recognized species and of these, only 9 members are known to infect humans [5]. In the human oral cavity, commonly detected HHV members include herpes simplex (HSV-1 and less frequently HSV-2), varicella-zoster virus (VZV), Epstein–Barr virus (EBV), human cytomegalovirus (HCMV) and Kaposi’s sarcoma associated herpesvirus (HHV-8) [6,7]. Herpesviruses are ubiquitous, with HSV-1 as the most prevalent, affecting 67% of the global population [8]. The common mode of transmission is via respiratory droplets and nasal secretions.

All herpesviruses establish lifelong latent infections in the host and can reactivate sporadically [9,10]. For instance, after primary infection of HSV-1 in oral mucoepithelial cells, the virus travels retrogradely along the axon and to the cell body to establish latency in the trigeminal ganglia [11]. Reactivation of latent herpes infections in the oral cavity may occur spontaneously or in response to stressors, including fever, UV light exposure, inflammation, or tissue injury [10]. Immunosuppression particularly renders individuals vulnerable to herpetic reactivation. Hence, the immunosuppressive state associated with COVID-19 may be a contributing factor to the reactivation of opportunistic pathogens [12,13,14]. Conversely, a recent pilot study suggests that immune dysregulation due to HHV infection, primarily EBV infections, may contribute to severe COVID-19 and the formation of post-COVID sequelae [15]. However, further investigation is needed to establish a bidirectional interaction between HHV and SARS-CoV-2.

Herpesviruses and SARS-CoV-2 are individually associated with oral inflammatory diseases, particularly periodontitis, peri-implantitis, and endodontic disease [16,17,18]. Herpesvirus and, more recently, SARS-CoV-2 are recognized as major etiological factors of the immune dysregulation and tissue destruction observed in all three diseases [19,20,21,22]. Given the established associations between systemic viral infections and oral inflammatory diseases, examining the potential association between COVID-19 and oral disease manifestations is pertinent. This review provides a synopsis of the reported associations between two trophic and highly transmissible oral viruses (viz., HHV, and SARS-CoV-2) and oral inflammatory diseases and expounds upon the relationship between systemic viral infection and oral disease.

## 2. Human Herpesviruses and Oral Inflammatory Diseases

Shindell et al. demonstrated the earliest evidence of herpesviruses’ presence in the oral mucosa. Viruses were detected in both chronic periapical and subacute granulomas, but their exact role remained unknown [23]. Later, his group successfully cultured viruses from the fragments of the oral mucosa (labial region) collected from subjects with recurrent herpes simplex. Tissue suspensions were directly inoculated onto primary human amnion and HeLa cell cultures. Herpes simplex virus was detected from some cultures by using neutralization tests containing a rabbit anti-herpes simplex serum, indicating the presence of other HHV members in infected cell cultures [24]. While the virus was readily detected in cell cultures inoculated with active lesions, the virus was not recovered from oral mucosa specimens obtained during the quiescent period, suggesting a latent infection in these sites. These studies provided basic information on the presence of HSV and other HHV in oral lesions but also suggested that healed lesions correlate with viral latency. Questions regarding the specific detection of HHV members in the oral tissues remained unanswered for a long time.

**Figure 1:**
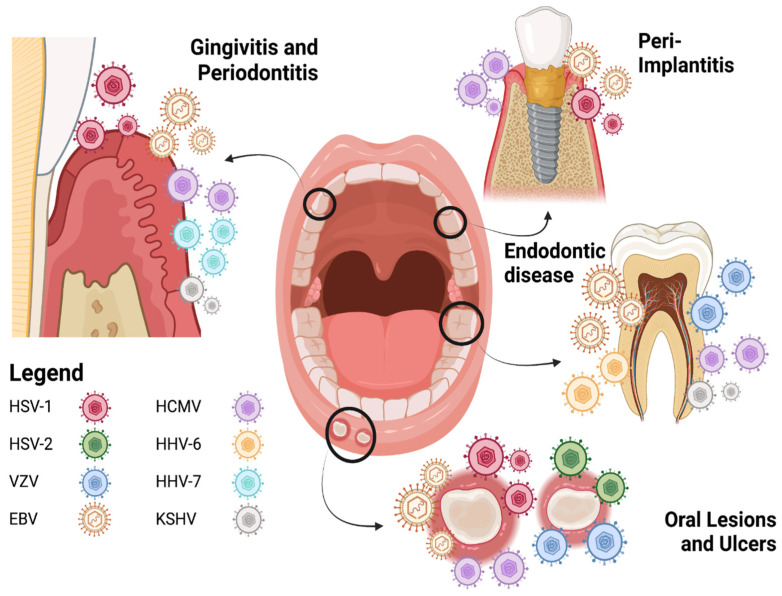
Human herpesviruses in oral disease. Larger and more plenteous virus depictions represent more prevalent or significant viral associations with disease. Created with Biorender.com (accessed on 1 November 2023).

Pioneering studies by Slots et al. rejuvenated the herpesvirus pathobiology in oral diseases [25]. Using PCR-based assays, Slots et al. provided evidence of the coexistence of multiple HHV members in different oral lesions. In particular, multiple studies reported a high prevalence and density of human herpesviruses in chronic and aggressive gingivitis and periodontal disease (Figure 1, Table 1), including HHV-7, EBV, HCMV, KSHV, HSV-1, and HHV-6B [4,26,27,28,29,30,31]. These findings are not surprising, as the infiltration of latent HHV-infected immune cells in inflamed oral tissues can increase viral titers locally and may provide a favorable microenvironment for HHV reactivation [4,8,32,33,34,35,36]. In addition, neurotrophic alphaviruses can reach oral tissues via the retrograde route. To facilitate the simultaneous detection of multiple viruses in the oral cavity, multiplex PCR assays have been increasingly utilized to detect oral pathogens, including herpesviruses and SARS-CoV-2 [37,38,39,40,41,42]. Research since the 1990s has led to the realization that herpesviruses may contribute to the pathogenesis of endodontic, periodontal, and peri-implant diseases. In the following sections, we will summarize our current understanding of HHV genotypes frequently detected in various oral tissues in health and disease.

### 2.1. Human Herpesviruses and Periodontal Diseases

Periodontal diseases, such as gingivitis and periodontitis, are infectious and inflammatory diseases that lead to the progressive destruction of dental-supportive tissues. Clinical symptoms include periodontal ligament and alveolar bone loss, color change, swelling, bleeding, gingival pain, and tooth mobility [43]. Periodontal disease involves complex interactions of infectious agents and host defense, as well as the interrelationship between the pathogens [44]. Herpesviruses are frequent inhabitants of oral tissue. However, their presence and periodontal pathogenicity in the periodontitis lesions are still unclear [45]. Studies from the Slots lab established a new periodontal infectious agent, herpesviruses, which were consistently detected in inflamed gingival biopsies and GCF. Viral genomes and transcripts of multiple HHV were identified at a higher prevalence in various cohorts. Multiple HHV members (including HSV-1, HCMV, EBV, KSHV, and HHV-6B) were routinely detected from gingival biopsy or GCF from the same diseased site [25,46]. In GCF collected from advanced periodontitis (AP; *n* = 30) and gingivitis (*n* = 26) subjects, 71% of samples were positive for at least one virus in the AP group. In comparison, only 31% samples were positive in the gingivitis group [25]. These findings suggest that the detection of HHV correlates with the inflammatory status of tissues. In immunocompromised HIV+ subjects, higher titers of HHV were detected, suggesting their pathogenic role in periodontal inflammation in immunocompromised/immunosuppressed hosts [47].

HHV (HCMV, EBV-1/2, HSV-1, HHV 6, HHV-7, and KSHV) genome detection in gingival biopsies from HIV-patients (*n* = 21) and non-HIV-patients (*n* = 14) showed a rich diversity of HHV members (at least 4–6) that showed a higher prevalence in HIV-positive subjects. KSHV was only detected in the gingiva of HIV-positive individuals. Importantly, it showed a strong correlation with various periodontal pathogens. EBV-1 and CMV were statistically associated with probing depths ≥ 6 mm and consistently detected in periodontal disease. Contreras et al. also evaluated the cellular tropism of HHV in gingival biopsies collected from healthy and periodontally diseased subjects (*n* = 20). Gingiva-derived single-cell suspensions were examined for viral protein and cell surface markers [48]. Many samples (70% cell fractions) showed the presence of targeted HHV. The myeloid cell compartment showed the presence of HCMV in 55% of cell fractions; T lymphocytes were positive for HCMV and HSV-1 (both 20%), and B cells were positive for EBV (45%). These findings provided a better understanding of cellular reservoirs of different HHV in vivo. However, further studies are needed to validate these findings to examine the status of viral activity in these cell type and how that impairs biological functions of key antiviral functions in the infected cells.

#### Human Herpesviruses and Bacterial Co-infection in Periodontal Diseases

In a recent study, HHV and bacterial (*P. gingivalis*, *T. forsythia*, and *P. intermedia*) association was examined in subjects diagnosed with periodontal abscess (PA; *n* = 39), necrotizing ulcerative periodontitis (NUP; *n* = 33), chronic periodontitis (CP; *n* = 27), and healthy (*n* = 30) periodontal tissue [30]. Compared to the control group, significantly higher detection rates of EBV and HCMV were reported in all diseased (PA, NUP, or CP) cohorts. This increase in HHV titers correlates strongly with an accumulation of *Pg*, *Tf*, and *Pi*. In particular, severe periodontal tissue destruction with deeper pockets correlates with CMV infection, suggesting viral–bacterial synergy in inflamed gingiva. Blankson et al. showed that HHV were exclusively detected in patients clinically diagnosed with aggressive periodontitis, implying that the inflammation status of periodontal tissues may impact viral accumulation or reactivation [27]. However, further research is needed to elucidate the consequence of viral infection on periodontal pathogenesis and severity.

A few studies have highlighted a less significant association between herpesviruses in periodontitis and a consistently stronger correlation between bacteria and periodontitis. While overall gingival HHV levels were not significantly different in HIV-positive and negative subjects, EBV-1 and co-infection (EBV-1–HCMV) were associated with HIV-positive patients with periodontitis [49]. Similarly, Emecen-Huja et al. reported that EBV was detected more frequently in sites containing deep periodontal pockets, but EBV and CMV levels were not associated with disease progression sites [50]. In contrast, an association was found between Gram-negative anaerobic bacteria and sites of disease progression. It is likely that a unique set of stimuli is required for the local accumulation of specific HHV genotypes. Dissecting molecular mediators of HHV and periodontal disease will unravel the relationship between the two distinct pathogens.

### 2.2. Human Herpesviruses and Peri-Implantitis

Peri-implantitis is characterized by the inflammation of soft or hard tissues surrounding a dental implant, creating mucositis and peri-implantitis. Its main clinical characteristics include the presence of erythema, bleeding on probing, swelling, and suppuration. Other symptoms include increased probing depths, the recession of the mucosal margin, and loss of alveolar bone [51]. Poor plaque control is the most common etiological factor for peri-implant disease, followed by dental implant mechanical problems and medical diseases. Global estimates suggest that 16% to 28% of implants fail, even higher in subjects with systemic conditions like diabetes, obesity, and hypertension [52]. As in periopathogenesis, microbial dysbiosis and immune dysregulation are critical factors in the development and progression of peri-implantitis. Biofilm removal helps with peri-implantitis prevention and management [53].

The etiology of peri-implantitis lesions can be associated with periodontal virus infection. Multiple studies have examined the prevalence of herpesvirus, including EBV, HHV-6, HHV-7, HSV-1, HSV-2, HCMV, VZV, and HHV-8 in peri-implantitis. Peri-implant plaque collected from 25 mucositis, 30 peri-implantitis, and 25 healthy peri-implant sites was examined for EBV and HCMV genotypes by PCR. Compared to healthy sites, which barely showed the presence of either viral genome, there was a high prevalence of HCMV-2 (53.3%) and EBV-1 (46.6%) in the peri-implant tissue plaque, suggesting a role of the viruses in the pathogenesis of peri-implantitis [54].

Another study evaluated the association of EBV and ten different periodontal pathogens—*Aggregatibacter actinomycetemcomitans* (Aa), *Candida albicans* (Ca), *Campylobacter rectus* (Cr), *Eikenella corrodens* (Ec), *Fusobacterium nucleatum* (Fn), *Porphyromonas gingivalis* (Pg), *Prevotella intermedia* (Pi), *Parvimonas micra* (Pm), *Treponema denticola* (Td), and *Tannerella forsythia* (Tf)—with peri-implantitis in plaque samples collected from 113 healthy and 77 peri-implantitis sites [55]. This study detected an accumulation of bacteria (including Tf, Pm, Fn, and Cr) in plaque, indicating a positive correlation between these bacteria and peri-implantitis. Moreover, Pi and Cr loads were detected in EBV-positive subjects, suggesting that the presence of EBV may exacerbate disease pathogenesis or severity. However, this study concluded that EBV levels did not correlate with peri-implantitis. This lack of correlation could be due to differences in sampling. Moreover, instead of tissue biopsies, this study examined EBV in microbial biofilm-rich plaque containing some desquamated epithelial cells or gingival-sulcus-associated immune cells. It is important to note that, unlike bacteria, viruses are obligate parasites, and viral detection in saliva, GCF, or tissue biopsies may yield reproducible results.

#### Human Herpesviruses and Bacterial Co-Infection in Peri-Implantitis

The HSV-1 and periopathic bacterial (Tf, Pm, Fn, Cr) association with peri-implantitis was assessed in the plaque samples collected from peri-implant sulcus and internal implant connections [56]. In a cohort of healthy and peri-implantitis subjects (*n* = 40/group), PCR data showed significantly higher HSV-1 titers. Interestingly, HSV-1-positive healthy and diseased sites show a differential association with periodontal pathogens. HSV-1-positive sites correlate with Tf, Pm, Fn, and Cr, while HSV-1-negative sites correlate with higher Aa, Pi, and Pm titers. A case report by Takahama et al. presented an association between EBV-positive mucocutaneous ulcers (EBV-MCUs) and systemic lupus erythematosus around dental implants [57]. The diagnosis of EBV-MCUs was based on clinical, radiographic, microscopic, and immunophenotypical features. EBV was detected in the oral lesion, suggesting an increased risk of viral etiology in autoimmune diseases.

How HHV may perturb local immune responses in peri-implantitis is a critical question. To address this, Marques Filho et al. collected saliva samples from subjects with implant placement (*n* = 21/group; healthy versus peri-implantitis groups) [58]. Afterward, they evaluated HHV genotypes and cytokines (IL-1β, IL-2, IL-4, IL-6, MCP-1, MIP-1α, and MIP-1β) in the samples. They observed a two-fold higher prevalence of HHV in the peri-implantitis group. Most of the cytokines tested did not show significant differences between the two groups; however, MIP-1β and TNF-α levels exhibited a positive correlation with the HHV-positive peri-implantitis group. These findings support the notion that immunomodulatory HHV may dysregulate pro-inflammatory cytokines.

Together, these studies suggest that the accumulation of herpesviruses in diseased sites may cause dysbiosis, likely by immune impairment, and contribute to the clinical manifestation of peri-implantitis. Although HHV prevalence is a unifying concept in oral inflammatory diseases, inconsistent sampling methods, the detection techniques used, smaller cohort size, and their significant inherent heterogeneity has shown contrasting results with varying levels of correlation among different studies.

### 2.3. Human Herpesviruses and Endodontic Diseases

Endodontic diseases are inflammatory oral infections of the dental pulp. Pain is the most frequent symptom of this pathology. Multiple microbial infections caused by the disruption of enamel integrity or periapical contamination can reach the root canal system. Infection may lead to inflammation of the pulp and periradicular tissues, which are classified as pulpitis and apical periodontitis [59]. However, these pulp infections can also be due to endodontic–periodontic lesions, which are pathological communications between the pulpal and periodontal tissues [60].

Multiple studies have examined the association between HHV infection and endodontic diseases. Inflammation of periapical, peri-radicular, or pulp tissue can disseminate signals for HHV reactivation and may cause the local accumulation of HHV members. HHV could play a role in the manifestation of inflammation by suppressing immune activity, resulting in a microbial activity that ultimately results in more damaging inflammation centralized in the endodontic tissues. Initial studies by Slots et al. showed the presence of HHV family members in inflamed pulp and disease periapical tissues [61,62]. Using PCR-based methodology, EBV, HCMV, and HSV-1 transcripts were quantified as symptomatic (*n* = 7) and asymptomatic (*n* = 7) periapical lesions [61]. HCMV and EBV were detected in seven (100%) and six (83%) symptomatic lesions, while only one sample was positive for either HCMV or EBV in asymptomatic lesions. HSV-1 was detected in only one asymptomatic lesion, suggesting a less significant contribution to periapical disease. Consistent with this, Slots et al. reported a higher prevalence of HCMV and EBV infections in symptomatic (*n* = 25) and asymptomatic (*n* = 19) periapical lesions as assessed by viral transcript quantification [62]. A higher frequency of HCMV was observed in symptomatic (100%) than asymptomatic periapical lesions (37%). Similar to the previous finding, all HCMV-positive lesions were also EBV-positive. A follow-up study quantified the presence of HCMV via flow cytometry and found 67% of periapical lesions were HCMV-positive. To examine HCMV tropism, they examined HCMV proteins in various immune subsets including monocyte/macrophage, T-cell, and B-cell surface markers [63]. HCMV was detected in monocytes and T cells but not in B cells. Overall, these studies laid the foundation for the future examination of herpesviruses in endodontic disease pathogenesis or exacerbation.

#### 2.3.1. Human Herpesviruses Activity and Endodontic Treatment Outcomes

Guilherme et al. examined the presence of different HHV (HSV-1/2, EBV, HCMV, HHV-6, and KSHV) in subjects’ saliva one-year post-root-canal treatment [64]. Among these, 45 subjects showed healed periradicular tissues, while 27 subjects presented with post-treatment apical periodontitis. EBV, HCMV, HHV-6, and KSHV were detected in all the samples to varying frequencies, while HSV-1/2 could not be detected. The prevalence of none of the viruses was significantly different in the two groups, suggesting that HHV presence is not correlated with the success or failure of the endodontic treatment. However, this study only examined one-year post-treatment time points, which might be long enough for viruses to attain latency. These findings suggest that periapical biopsies are better for evaluating HHV correlation with treatment outcomes.

To examine viral detection and activity, simultaneous quantification of viral DNA and mRNA can better assess viral prevalence. In samples collected from patients with irreversible pulpitis (*n* = 29) or apical periodontitis, either primary (*n* = 30) or previously treated (*n* = 23), the DNA and mRNA of different HHV (HSV-1, HCMV, EBV, and VZV) was quantified by using primary and nested PCR [65]. EBV DNA and mRNA were detected in 43.9% and 25.6% of diseased lesions, respectively, while none of the healthy controls showed viral presence. The HSV-1 genome was detected in 13.4% of periapical lesions but not in control tissues. Intriguingly, HCMV was detected at a higher frequency in healthy samples than in diseased cohorts. HCMV DNA and mRNA were detected in 42.1% and 10.5% of healthy pulps, respectively, while diseased lesions showed a 15.9% and 29.3% prevalence rate for viral DNA and mRNA. Hernádi et al. (2013) quantified the EBV and HCMV genome and mRNA in periapical lesions. PCR results showed that EBV was more prevalent than HMCV in the biopsies [66]. Compared with healthy controls (2.5%), diseased tissues showed a 72.5% and 50% prevalence (significantly higher) for the EBV genome and mRNA, respectively. HCMV was detected only in 10% of diseased tissues; however, the virus was not detectable in healthy tissues. A recent study examined EBV and HCMV levels in apical periodontitis lesions (*n* = 100) using two different PCR methods: nested primers and Taqman probes. Among the tested biopsies, 12 of 74 samples (16.2%) were EBV-positive, and 4 of 54 (13.5%) tested samples were HCMV-positive [67]. Thus, HHV prevalence likely varies with cohorts and detection methods. However, these studies did not observe any association between HHV presence and symptomatic/asymptomatic lesions. These results were contrary to the findings of Verdugo et al., which showed a significant correlation between symptomatic periapical periodontitis lesions and HHV presence [68]. Total genomic DNA was isolated from asymptomatic/symptomatic periapical periodontitis lesions and unstimulated saliva to detect EBV, HCMV, and periopathic bacterial genomes by PCR. Compared with asymptomatic lesions, symptomatic lesions showed a higher accumulation of periodontal bacteria (*Porphyromonas gingivalis*, *Aggregatibacter actinomycetemcomitans*, *Prevotella intermedia*, and *Treponema denticola*). In addition, they were ~4 times more likely to be infected with EBV. This study was consistent regarding HHV prevalence in periapical lesions but also showed an association between higher HHV levels and symptomatic lesions and periopathic bacterial presence.

To examine the role of EBV in endodontic pathosis, Makino et al. performed RT-qPCR of the EBV viral genome, in situ hybridization (ISH) of EBV-encoded small RNA (EBER, a viral noncoding RNA), and immunohistochemical (IHC) staining for LMP-1 in surgically removed periapical granulomas (*n* = 2) [69]. PCR analysis identified that ~70% (25/30) biopsies were EBV-positive. Consistent with PCR data, ISH and IHC showed EBER and LMP1 staining, respectively, in B cells/plasma cells in six out of nine (~67%) biopsies. These results show that the viral genome, protein, and RNA are frequently detected in inflamed periapical tissues. EBER and LMP1 are expressed predominantly in the latent phase. Supporting this notion, only B cells were found to be positive for probes against EBER. However, the data presented here do not resolve the issue of whether EBV is active or largely latent in inflamed tissues. In addition, whether similar observations hold true for other HHV members’ remains to be determined.

#### 2.3.2. Human Herpesviruses and Endodontic Diseases in Immunocompromised Patients

HHV reactivation in immunocompromised patients is highly common [43,47,70]. To assess if HHV is recruited to inflamed endodontic tissues in immunocompromised humans, Saboia-Dantas et al. performed an immunohistochemical analysis of periapical lesions collected from 35 patients [71]. Of the periapical lesion biopsies, 31% and 23% were positive for EBV and HCMV, respectively, while 14% of the lesions were dual-positive for EBV and HCMV. A higher seroprevalence of both HCMV (67%) and EBV (44%) was observed in periapical lesions from HIV-positive subjects, compared to HIV-negative (HCMV = 7% and EBV = 27%), suggesting that endodontic tissues are susceptible to viral reactivation in immunocompromised subjects.

In a systematic analysis, Jakovljevic and Andric examined HCMV and EBV accumulation on the etiopathogenesis of apical periodontitis [72]. HCMV and EBV were often identified in periapical lesions. However, a significant relationship between HCMV and EBV detection and clinical features of apical periodontitis was not observed. While the genomic detection of HHV is vital for association analysis, only limited studies have examined the active status of HHV. This knowledge gap is significant because latent HHV-infected cells can accumulate in diseased oral tissues; however, the exact pathogenic role of the HHV can only be assessed with information on the virus activation.

## 3. SARS-CoV-2 and Oral Inflammatory Diseases

Accumulating evidence suggests that the oral cavity is a critical player in SARS-CoV-2 infection and severity [73,74]. To enter the human body, SARS-CoV-2 uses its spike protein (S protein) to bind to the angiotensin converting enzyme II (ACE2) receptor, which is highly expressed in the salivary glands of the oral cavity, cellular membrane of mucosal tissue cells in the tongue, surrounding buccal, gingival tissues, and pulp [18,75,76]. Recently, SARS-CoV-2 was identified in gingiva and gingival crevicular fluid (GCF) [77]. Together, these observations reveal that oral tissues are trophic for the virus. Consistent with this, clinical oral manifestations in COVID-19 patients range from ulcers, blisters, white and erythematous plaque, necrotizing gingivitis, salivary gland alterations, tongue depapillation, and gustatory dysfunction (Table 2) [21,78,79,80,81,82,83,84,85,86]. Importantly, multiple studies report long-term oral cavity symptoms following COVID-19 infection. These symptoms are collectively referred to as oral post-acute sequelae of COVID-19 (PASC) and include erythematous bullae on the palate, non-bleeding lesions in the palatal mucosa, tongue enlargement, and burning mouth [59,87]. In a clinical case study by the clinic “Stomatologia Rafałowicz”, out of the 1256 studied patients, 68% of patients displayed oral symptoms: discoloration, ulceration, and hemorrhagic changes on the oral mucosa (32%); mycosis on the tongue (29.69%), unilateral aphthous-like lesions on the hard palate (25.79%), and atrophic cheilitis (12.5%). These patients were diagnosed with COVID-19 2 to 6 months before oral examination [19]. Oral symptoms appear to be worsened and more prolonged in the elderly with coexisting systemic diseases and patients with severe COVID-19 [19,88].

Drivers of COVID-19-related symptoms in the oral cavity include stress, poor oral hygiene, multi-organ disorders, and notably, immune system dysfunction [19,21,22]. An improper immune response may allow for opportunistic infections. This scenario may explain why oral lesions in COVID-19 subjects appear concomitant with the hypergrowth of opportunistic oral pathogens, such as herpesviruses [59]. Recurrent herpetic lesions and higher bacterial growth in COVID-19 subjects suggest the dysregulation of immune responses that augment opportunistic co-infections (bacterial and viral). Painful oral lesions and ulcers were reported to affect keratinized and non-keratinized tissue in a manner similar to those caused by HHV in suspected and confirmed COVID-19-infected patients [84]. Intriguingly, the resolution and healing of oral lesions correspond to the overall resolution of COVID-19 symptoms, suggesting that COVID-19 and the oral manifestations were linked. SARS-CoV-2 and its interaction with ACE2 on epithelial keratinocytes could influence their function, resulting in the development of lesions and ulcers [79]. Ultimately, SARS-CoV-2 elicits an inflammatory immune response that varies in intensity and duration among infected individuals. Thus, the virus can manifest in various oral diseases or in other parts of the body, with differing clinical manifestations between infected individuals. The following section will discuss the link between SARS-CoV-2 and oral diseases such as periodontitis, peri-implantitis, and endodontic disease.

### 3.1. SARS-CoV-2 and Periodontal Disease

Recent studies have revealed an association between COVID-19 infection and periodontitis severity [89,90]. Clinical data indicate a positive correlation between periodontitis and COVID-19 complications. This relationship is evident in a case–control study by Marouf et al., which used the national electronic health records of the state of Qatar between February and July 2020. Compared to COVID-19 patients with early-stage or no periodontitis, the study found that COVID-19 patients (*n* = 528) with moderate-to-severe periodontitis had increased odds for ICU admission (OR = 3.54, 95% CI 1.39–9.05), need for assisted ventilation (OR = 4.57, 95% CI 1.19–17.4), and death (OR = 8.81, 95% CI 1.00–77.7). Marouf et al. also found significantly higher levels of D-dimer, WBC, and C-reactive protein (CRP) in the serum of COVID-19 patients with periodontitis than those without periodontitis. This result is significant because elevated D-dimer, WBC, and CRP blood levels are considered biomarkers of worsened COVID-19 prognosis, suggesting that periodontal disease could exacerbate COVID-19 clinical outcomes [83].

#### 3.1.1. Mechanistic Link between SARS-CoV-2 and Periodontal Disease

Several hypothetical mechanisms explain the link between COVID-19 and periodontitis: (1) the elevated T helper (Th)-17 response in severe periodontitis amplifies a ‘cytokine storm’, which recent studies associated with worse prognosis and increased mortality rate due to COVID-19, (2) periodontal pockets serve as an ideal reservoir for SARS-CoV-2, (3) periodontopathic bacteria increase ACE2 expression and promote inflammatory cytokine production in the lower respiratory tract via food or salivary aspiration, (4) periodontopathic bacteria enhance the infectivity of SARS-CoV-2 by producing proteases that cleave the S protein, and (5) increased Gal-3 expression in severe periodontitis enhances Gal-3-mediated immune response and viral attachment [17,83,91,92,93,94]. Given the established link between periodontitis, a chronic inflammatory disease localized in the oral cavity, and other systemic inflammatory diseases in the existing literature, understanding the relationship between COVID-19 and periodontal disease manifestation and aggravation is critical to effectively treating oral inflammation [95,96,97]. Below, we will discuss each hypothetical mechanism in detail, starting with the role of periodontopathic bacteria in COVID-19 pathogenesis.

**Figure 2:**
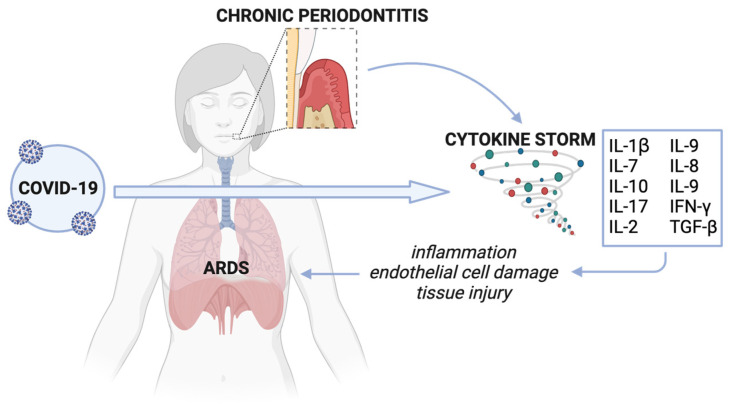
Cytokine storm associated with chronic periodontitis and COVID-19. Cytokines resulting from untreated chronic periodontitis disseminate intravascularly and contribute to COVID-19-associated cytokine storm. This aggravated cytokine storm worsens systemic inflammation, causing widespread tissue damage, including in the capillary and endothelial epithelium, ultimately resulting in ARDS. Created with Biorender.com (accessed on 26 December 2023).

Non-resolving inflammation triggered by a dysbiotic subgingival biofilm is characteristic of chronic periodontitis. This localized chronic inflammation can subsequently translate to systemic inflammation and the elevated production of inflammatory markers in circulation. In particular, chronic periodontitis often involves an IL-17/Th17-mediated pro-inflammatory response—a significant contributor to cytokine storms. Cytokine storms are characterized by an excessive release of inflammatory molecules into the bloodstream and result from the hyperactivation of immune cells, such as lymphocytes and macrophages, which primarily produce cytokines. Similar overexpression of several cytokines (IL-1β, IL-7, IL-10, IL-17, IL-2, IL-9, IL-8, IL-9, IFN-γ, TGF-β, and some metalloproteinases) is seen in chronic periodontitis and COVID-19 [83,93,94,98]. Mounting evidence suggests an association between cytokine storms and COVID-19 severity, particularly with the development of acute respiratory distress syndrome (ARDS), a life-threatening condition characterized by fluid build-up in the alveoli seen in some COVID-19 patients, resulting in hypoxemia and multi-organ failure (Figure 2) (Ragab et al., 2020). Due to a similar cytokine storm profile between chronic periodontitis and COVID-19, Sahni et al. suggest that the robust IL-17/Th17 response in chronic periodontitis could aggravate the cytokine storm seen in COVID-19 [83,93].

#### 3.1.2. Peiodontital Tissue as a Target for SARS-CoV-2 Infection

As periodontitis progresses, tissue damage, detachment of junctional epithelium, and chronic inflammation contribute to the formation and widening of periodontal pockets, which refer to abnormally deepened gingival sulcus (>3 mm). Periodontal pockets harbor dysbiotic subgingival plaque biofilms and viruses, such as bacteriophages and herpesviruses. In the case of SARS-CoV-2, a study by Badran et al. hypothesized that periodontal pockets could serve as plausible reservoirs for the virus. Moreover, these pockets could facilitate SARS-CoV-2 replication and migration to systemic tissues via the capillary periodontal complex [91]. Possible SARS-CoV-2 entry points into the periodontium involve the S protein–ACE2 and S protein–CD147 interactions. Multiple studies have shown the binding of S protein to ACE2, which Santos et al. confirmed it as expressed in human periodontal ligament fibroblasts [99]. Less studied, however, is CD147 and its possible role as a novel, non-canonical viral receptor for SARS-CoV-2. Wang et al. showed that administering a CD147-blocking antibody on Vero E6 and BEAS-2B cells inhibits SARS-CoV-2 amplification [100]. Previous studies have shown that CD147 is expressed in periodontal pockets, particularly in the buccal and subgingival regions [91,101]. Additionally, post-mortem data from Matuck et al. confirm the presence of SARS-CoV-2 in the periodontal tissue of COVID-19-positive patients [102]. Together, these findings suggest that periodontal tissue is a target for SARS-CoV-2 infection, particularly in cases of chronic and aggressive periodontitis.

#### 3.1.3. SARS-CoV-2 and Periodontopathic Bacteria

When food and saliva are unintentionally aspirated, periodontopathic bacteria can migrate into the lower respiratory tract [103,104,105]. Previous studies have identified periodontopathic bacteria in the bronchoalveolar lavage fluid of COVID-19 patients. Takahashi and Watanabe et al. hypothesize that bacterial colonization in the lungs and bronchi can promote SARS-CoV-2 infection by releasing pathogenic factors, such as endotoxins, which increase ACE2 expression [94]. Additionally, these bacteria stimulate the production of pro-inflammatory cytokines in the lower respiratory tract, such as IL-6 and IL-8, thereby promoting COVID-19 aggravation. To support this stance, Takahashi and Watanabe et al. demonstrate that the culture supernatant of the periodontopathic bacteria Fusobacterium nucleatum upregulates ACE2 expression and IL-6 and IL-8 production in human alveolar epithelial cells. The authors observed a similar increase in IL-6 and IL-8 in other human epithelial cells, including BEAS-2B bronchial and Detroit 562 pharyngeal epithelial cells [94].

Another possible mechanism by which periodontopathic bacteria enhance SARS-CoV-2 tropism is by producing proteases that cleave the S protein. After viral entry, proteolytic cleavage of the S protein at the S1/S2 and S2′ cleavage sites are necessary to mediate viral attachment to host cells and viral–host membrane fusion. Specifically, the host proprotein convertase furin cleaves at the S1/S2 site, while the transmembrane serine protease 2 (TMPRSS2) cleaves at the S2′ site. In vitro data show that treating Calu-3 human airway epithelial cells with combined TMPRSS2 and furin inhibitors displays potent antiviral activity against SARS-CoV-2 proteolytic activation and replication [106]. Similarly, proteases produced by periodontopathic bacteria have been shown to cleave hemagglutinin (HA) into HA1 and HA2 during influenza virus infection. Takashi and Watanabe et al. suggest that these bacterial proteases could play the same role during SARS-CoV-2 infection, although further experimentation is needed to confirm this hypothesis [94].

Kara et al. suggest that increased Galectin-3 (Gal-3) levels are positively associated with periodontitis severity, and this relationship could explain the link between periodontitis and COVID-19. Gal-3 is a widely expressed pro-inflammatory protein and a key regulator component of immune cell homeostasis, which it modulates by controlling T-cell-mediated inflammation. Inhibition of Gal-3 reportedly decreases the production of the pro-inflammatory cytokines IL-1 and IL-6 while increasing the production of the anti-inflammatory cytokine IL-10 [92,107]. Gallo et al. reported higher plasma levels of Gal-3 in patients with severe COVID-19 compared to healthy controls [108]. In terms of structure, a component of SARS-CoV-2′s S protein (i.e., S1-N terminal domain) and Gal-3 are strikingly similar. This resemblance is significant because, in other viral diseases such as HIV and HTLV, Gal-3 reportedly serves as an attachment factor that mediates viral entry into T-cells [109]. Therefore, the Galectin-like S1-NTD is believed to increase the SARS-CoV-2 immune response and viral attachment to host cells [92]. While the previously mentioned articles described the observed relationship between periodontitis, its associated pathogens, and COVID-19 infection and severity, few articles address how COVID-19 affects periodontitis. Recently, oral examinations of patients infected with SARS-CoV-2 report the presence of uncharacterized periodontal lesions, specifically necrotizing periodontal disease (NPD). NPD is a type of periodontal disease common among HIV and immunocompromised patients. It is characterized by necrosis in the alveolar bone, gingival tissues, and periodontal ligament. Metagenomic analyses of SARS-CoV-2 patients detected an increased prevalence for oral disease-implicated bacteria, including *Prevotella intermedia*, *Fusobacterium* spp., and *Veillonella* spp. [85,110]. Interestingly, another study examining the relationship between the nasopharyngeal microbiome and COVID-19 discovered decreased Fusobacteria presence in patients with COVID-19 [111]. Of note, *P. intermedia* and *Fusobacterium* spp. are frequently associated with NPD [112]. Bacterial co-infections with SARS-CoV-2 may predispose individuals to NPD [113]. Therefore, it is crucial to not only study how oral health affects SARS-CoV-2 infection and severity but to also examine how SARS-CoV-2 can lead to altered oral disease manifestation.

### 3.2. SARS-CoV-2 and Peri-Implantitis

Peri-implantitis is characterized by the bacteria-induced destructive inflammatory process affecting the implant-surrounding hard and soft tissues. Similarities between peri-implantitis and periodontitis suggest an association between SARS-CoV-2 and peri-implantitis. The clinical manifestations and cellular processes shared by peri-implantitis and periodontitis include alveolar bone loss, decreased osseointegration, increased pocket depth, and bleeding on probing [114]. Moreover, peri-implant and periodontal infections share an analogous sequence of inflammatory events and qualitative composition of immune cells (i.e., predominant neutrophils, macrophages, T cells, and B cells). Nonetheless, fundamental differences exist between the two oral diseases. Relative to periodontitis, peri-implantitis involves a higher proportion of immune cells and associated inflammatory mediators. Compounding this larger inflammatory infiltrate is the absence of periodontal ligament and Sharpey’s fibers around implants, resulting in less vascularization and connective tissue attachment, respectively. Altogether, these differences can help to explain the more severe and rapid progression of tissue destruction in peri-implantitis compared to periodontitis [115].

These immunopathological events necessitate the question: does peri-implantitis affect COVID-19 infectivity and severity? Unfortunately, there remains a tremendous gap in knowledge in the literature. While most research focuses on the association between SARS-CoV-2 and periodontitis, with the increasing prevalence of peri-implant diseases, the same attention should be paid to elucidating the link between SARS-CoV-2 and peri-implantitis.

### 3.3. SARS-CoV-2 and Endodontic Disease

A higher percentage of endodontic emergencies in the post-pandemic period compared to the pre-pandemic period further suggests an association between virus infection and the exacerbation of oral diseases. A retrospective survey of ~2500 patients (2–92 years; mean age 38.9 ± 19.3 years) in China indicated a marked increase in the proportion of dental and oral infections [85,116,117]. Yu et al. reported that an increase in irreversible pulpitis was the most common pathology observed in a cohort of ninety-six subjects (mean age 42.24 ± 18.32 years) from Wuhan, China. In this study, SARS-CoV-2 confirmed or suspected subjects reported significantly higher pain levels, suggesting more severe disease manifestation [117].

Though bacteria (i.e., Streptococci and Staphylococci spp.) are the usual drivers of pulpitis, co-infection with viruses may play a critical role in the development of endodontic pathosis. Herpesviruses, specifically EBV, are associated with irreversible pulpitis [65]. In the case of SARS-CoV-2, research regarding the association between COVID-19 and pulpitis is sparse, as is the case for other endodontic diseases. A transcriptome-wide effect cross-analysis suggests that the dental pulp is susceptible to SARS-CoV-2 infection [18]. Galicia et al. showed that ACE2 and TMPRSS2 are expressed in the dental pulp, but whether endodontic diseases predispose individuals to SARS-CoV-2 infection remains unclear.

The potential underlying roles of SARS-CoV-2 in worsening clinical manifestations of oral diseases remain elusive. Perhaps SARS-CoV-2 infection (1) takes advantage of preexisting periodontal and endodontic diseases or (2) creates new avenues for extended tissue damage by enhancing the severity and aggressiveness of tissue degradation, resulting in intense pain and dentinal and cemental pathology. The underlying mechanisms of hyper-inflammation in COVID-19 subjects reflect the crosstalk between SARS-CoV-2 and oral diseases. Whether SARS-CoV-2 and oral virus/bacteria interact synergistically demands further investigation. Persistent inflammation of periodontal and endodontic tissues has been shown to affect systemic health and may facilitate crosstalk of the underlying operative mechanisms mediating an increase in the oral and systemic clinical symptoms of COVID-19 through common chronic inflammatory pathways [118,119]. To demonstrate the impact of the SARS-CoV-2 virus on oral health and its role in exacerbating oral infections, larger cohort studies comparing SARS-CoV-2-negative and positive subjects are necessary [59]. However, there is promising evidence that the management of good oral hygiene can potentially prevent COVID-19 aggravation [120].

## 4. Saliva as a Biological Fluid for Viral Diagnosis

**Figure 3:**
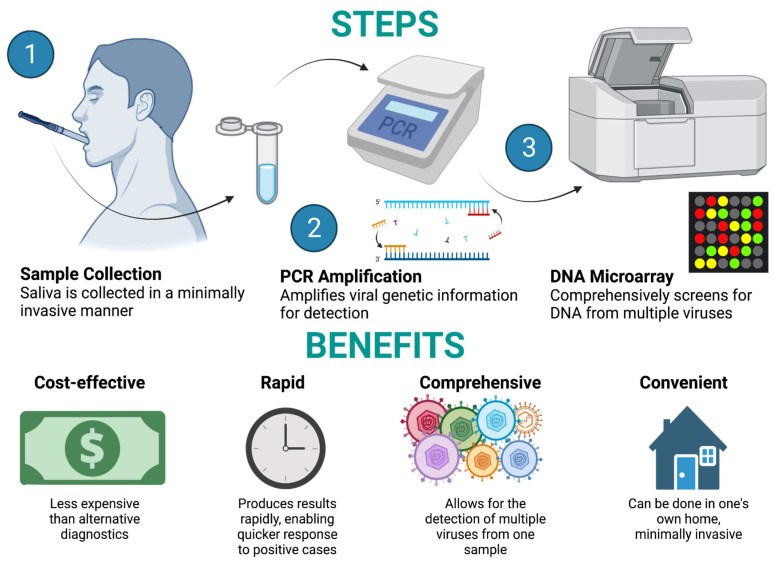
Saliva as a biological fluid. Steps for minimally invasive saliva sampling during herpesvirus and SARS-CoV-2 diagnostic testing. Created with Biorender.com (accessed on 29 November 2023).

Clinical professionals and scientists have often used saliva as a biological fluid for viral diagnosis and to characterize the microbial profile of the oral cavity. Alternatives to saliva tests include serum and plasma tests [121,122], as well as whole blood and urine tests [123]. Saliva tests have numerous benefits to other testing methods: they are less invasive, relatively inexpensive, and feature easily acquirable samples (Figure 3). In various cases and research studies, saliva tests were used (often followed by PCR to quantify and amplify viral DNA) to detect viral presence and activity [64,124,125]. In practice, saliva tests can efficiently and economically provide researchers and healthcare professionals with a clearer understanding of the state of viral infection in the oral cavity and throughout the body.

### 4.1. Advantages of Saliva-Based Diagnostics

Because saliva is one of the main transmission paths for herpesviruses in humans, saliva testing is a practical diagnostic method for herpesvirus detection [123]. Since many herpesviruses manifest themselves through oral and skin rashes and lesions, the virus has already multiplied undetected by the time patients report these lesions. By using saliva tests to screen for viral infection, viruses like varicella-zoster virus (VZV) can be preemptively detected at earlier stages in the viral infection process before any rashes or lesions appear [126]. In this way, viral detection precedes oral and physical manifestation, allowing for earlier treatment administration and less painful, more positive health outcomes. Through continuing advances in saliva testing procedures and technology, tests for viruses like VZV are becoming faster and more specific, with higher DNA yields that allow even earlier detection [126]. This early detection is critical to patient health outcomes because viral DNA like VZV found in saliva can act as a biomarker for diseases caused by these viruses, including postherpetic neuralgia (PHN) [127]. Thus, saliva tests allow researchers and providers to access information regarding viral infections in the body and oral cavity, even before they manifest themselves in physical symptoms. Subsequent early detection can allow for early and more effective treatment administration.

In addition, saliva testing allows for a more comprehensive look at viral activity in the body, including the subtle effects of viruses on the immune system. In a study examining herpes labialis lesions caused by herpes simplex virus-1 (HSV-1), saliva tests were used to observe the activity of inflammatory cytokines and HSV-1 secretion in human subjects [128]. That report cited cases in which herpesvirus lesions were treated with antiviral photodynamic therapy (aPDT), an alternative to traditional antiviral treatment that was more expensive and could result in viral resistance. Later, a randomized clinical trial revealed only marginal benefits on the first day of treatment [129]. Another article highlighted the potential benefits of using salivary tests to gather relevant information about viral activity and prevalence in the oral cavity, allowing for more accurate and relevant diagnoses. The authors touched on how saliva tests were used in patients with HIV presenting white lesions on the tongue, and saliva tests were administered to comprehensively search for herpesviruses in the oral cavity [130]. The saliva tests used PCR and a DNA microarray to measure the prevalence of various Herpesviridae including HSV-1, HSV-2, VZV, CMV, EBV, HHV-6, HHV-7, and HHV-8. Using a saliva test, the authors tested for many viruses at once, giving them a fuller picture of the oral viral activity while also doing so in a cost-effective and timely fashion. This testing method can give researchers and providers a fuller picture and a better understanding of what viruses may be present and active in a patient. With that information, providers can make more accurate diagnoses.

### 4.2. Saliva-Based Diagnostics and Self-Administered Testing

Saliva tests have also been used for self-testing at home, making valuable testing information accessible to even more people. An article from 2018 described the benefits of at-home testing kits for HIV and called for the implementation of more such tests [131]. A follow-up article in 2020 described the results of increased self-testing kit distribution: more men tested themselves without the challenges of going into a lab to receive testing [132]. Ultimately, these saliva testing kits led to increased access to testing in the target population. This example illustrates the broader benefits of saliva testing for viruses. By using saliva tests, more people can be tested less invasively, and more viruses can be screened for with high specificity. Monitoring viral infections can facilitate timely treatment, improving health outcomes and increasing public knowledge of viral prevalence in larger populations.

### 4.3. Advancements in Saliva-Based Diagnostics during the SARS-CoV-2 Pandemic

During the SARS-CoV-2 pandemic, healthcare professionals and scientists prioritized the development of rapid, accessible, affordable, and accurate testing to allow for better control of the spread of the virus. In addition to new policies designed to contain the spread of the virus, involving mask-wearing and physical distancing, viral testing quickly became a critical tool in the battle against SARS-CoV-2. Different diagnostic assays have emerged, each with benefits and drawbacks related to cost, accessibility, effectiveness, and convenience. Saliva tests are a particularly beneficial diagnostic tool in the monitoring of SARS-CoV-2 spread because of their minimally invasive and practical characteristics. Throughout the pandemic, saliva tests have provided affordable testing with timely and accurate results.

As mentioned above, Gabriel et al. described the benefits of at-home testing kits for HIV and called for the widespread implementation of similar diagnostic tests for improved viral screening [131]. A follow-up study concluded that testing rates were higher in men who tested themselves than those who faced the difficulties of going to a lab to receive testing [132]. The study aimed to determine whether administering free HIV self-testing kits would be realistically possible and cost-effective, and if so, whether increased use of these tests would translate to higher testing frequencies, more HIV diagnoses, and earlier clinical treatments. The researchers sought to diagnose a higher percentage of HIV-positive individuals in less time using at-home viral testing kits. The results of the study indicated that the subjects described saliva testing as convenient and comfortable. However, the two users who tested positive for HIV in the study questioned the validity of the test and sought additional testing in-clinic. In this study, seeking additional testing was a desirable result, since those who tested positive for the virus were motivated to seek additional help when they otherwise would not have.

### 4.4. Accurate Alternatives to Saliva-Based Diagnostics

Many saliva tests utilize a quantitative reverse transcription–polymerase chain reaction (RT-qPCR) to detect viral RNA in subject saliva samples [133]. The demand for SARS-CoV-2 diagnostic testing catalyzed the development of different sampling technologies that test for viral RNA using RT-qPCR, including nasal and pharyngeal swabs [134]. RT-qPCR testing is particularly beneficial due to its relatively low cost and high sensitivity [135]. While some have reported as much as 20% higher sensitivity with diagnostic assays utilizing nasopharyngeal swabs when compared to saliva samples, saliva samples provide broader testing accessibility and allow for more individuals to be tested in a more comfortable and minimally invasive manner [134]. Of note, self-administered nasopharyngeal and oral swabs, including those used in at-home testing kits, have been utilized in multiplex RT-qPCR analysis assays with an accuracy comparable to tests administered by healthcare professionals [40,41]. Altogether, the accessibility, comfortability, sensitivity, and affordability provided by RT-qPCR saliva diagnostic tests for SARS-CoV-2 have made saliva tests a staple in the arsenal of testing techniques to identify infection and combat viral transmission quickly.

## 5. Discussion

While bacteria and fungi are the traditional focus of oral disease etiology, the contribution of viruses to oral pathologies is gaining increasing recognition. Viral infections of the oral cavity are common; however, our understanding of their role in oral disease pathobiology remains understudied. This review highlights the role of herpesviruses (dsDNA virus), and SARS-CoV-2 (ssRNA virus) in chronic inflammation-driven oral diseases, particularly gingivitis, periodontitis, peri-implantitis, and endodontic disease. While herpesviruses often establish latency in the oral mucosa, where they can reactivate and cause recurrent episodes of inflammation, growing evidence suggests that the periodontal pocket, gingival sulcus, and dental caries lesions serve as reservoirs for SARS-CoV-2 [11,136]. We provide evidence strongly supporting a relationship between HHV and SARS-CoV-2 and different oral inflammatory diseases. Interestingly, a synergistic viral-bacterial interaction could explain the co-existence of selective periodontal bacteria in infected/inflamed oral tissues; however, the underlying molecular and cellular pathways remain understudied.

Because of the prevalence of viruses in the oral cavity and their contribution to inflammation-mediated tissue destruction, there exists a pressing need for viral diagnostic techniques. Modern testing strategies, such as saliva testing, provide accessible, minimally invasive testing that promotes overall health and curbs disease transmission, particularly in the case of SARS-CoV-2. As the COVID-19 pandemic has demonstrated, effective viral testing, coupled with an understanding of the association between prevalent viruses and oral disease manifestation, is critical for the accurate diagnosis and optimal treatment of oral inflammatory diseases. As the world’s collective knowledge surrounding viral infections grows through continued research, viral diagnostic techniques will continue to improve in affordability, accuracy, accessibility, and applicability. These advancements in viral detection technologies will provide increased access to viral testing and earlier diagnoses of viruses, resulting in improved oral and systemic health for all.

## 6. Future Directions

This review discusses the relevant associations of herpesviruses and SARS-CoV-2 with oral inflammatory diseases. However, a pressing need remains to examine the role of virus–bacterial and virus–virus interactions in propelling inflammation and its associated oral diseases. Publications from our lab and others review potential herpesviral–bacterial synergism in the context of oral disease [5,137,138,139]. For example, a pilot study by Verdugo et al. demonstrates that symptomatic periapical periodontitis patients with higher proportions of periodontopathogens—*Pg*, *Td*, *Pi*, and *Aa*—were 3.7 times more likely to be infected with EBV than asymptomatic periapical periodontitis patients [68]. The positive correlation between EBV and periodontopathogenic bacteria necessitates future experiments investigating the potential causative relationship between EBV, specific anaerobic oral bacteria, and symptomatic periapical periodontitis. One possible mechanism involves the uncontrolled release of proinflammatory cytokines (e.g., IL-1β and TNF-α) in response to herpesvirus infection, supporting the proliferation of periodontopathogens and resulting in tissue destruction [140]. Potential herpesviral–bacterial synergism extends to endodontic lesions. A study by Ferreira et al. reveals concurrent detection of herpesvirus and bacterial DNA in the majority of endodontic abscess samples [137]. Nonetheless, the exact role of herpesviral–bacterial cooperation in endodontic lesions remains unclear.

Literature regarding the role of SARS-CoV-2 and bacteria co-infection in oral disease is even more scarce. A meta-analysis study by Tan et al. demonstrates a drastic difference in the oral microbiome composition in COVID-19-positive patients compared to healthy controls, especially among the elderly [141]. Furthermore, a metagenomic analysis of COVID-19-positive patients revealed high reads of bacteria commonly implicated in oral disease (e.g., *P. intermedia*, Streptococci, Fusobacterium, Trepenome, and Veillonella) [110]. Finally, a case–control study by Patel et al. suggests that antibiotic and antiseptic treatments are effective against COVID-19 and oral disease symptoms. Here, after a 5-day course of metronidazole and a 10-day regimen of 0.12% chlorhexidine mouthwash, the patient with suspected COVID-19 reported complete resolution of oral symptoms and fever, supporting the potential role of bacterial co-infections in the severity of COVID-19 [85]. While these studies hint at a positive correlation between the population of SARS-CoV-2 and oral bacteria, there is a need to investigate the exact mechanisms by which these pathogens potentially cooperate to jumpstart or aggravate oral inflammatory diseases.

The investigation of potential interactions of SARS-CoV-2 with co-circulating herpesviruses and oral bacteria has the potential to yield valuable findings, especially given the prevalence of HHV infections in the oral cavity. After all, burgeoning evidence suggests that pathogens do not exist in isolation, and the interaction between multiple pathogens has significant implications for the progression of oral disease. Therefore, future research investigating the underlying mechanisms of SARS-CoV-2, human herpesvirus, and oral bacteria co-infection will provide needed insight into the diagnosis, treatment, and prevention of chronic oral inflammatory diseases.

Furthermore, continued advancements in viral diagnostic techniques, particularly nucleic-acid-based assays, will enable comprehensive, earlier detection of viral infection and potentially lead to improved treatment strategies for pathogen-driven oral inflammation. Developments in viral diagnostics have proven invaluable in global efforts to facilitate early diagnosis of SARS-CoV-2 infection and mitigate its spread, and multiplex RT-qPCR assays have grown in popularity due to their accuracy and cost-effectiveness, allowing for efficient viral RNA screening for multiple SARS-CoV-2 variants in a single test [142,143]. Multiplexing approaches using qPCR for viral RNA and DNA detection have been effectively utilized to detect a variety of pathogenic viruses besides herpesviruses and SARS-CoV-2, including respiratory viruses like influenza A, influenza B, respiratory syncytial virus (RSV), human metapneumovirus (HMPV), and human parainfluenza virus (HPIV); blood-borne pathogens such as retrovirus, adenovirus, enterovirus, and poxvirus species; and even viruses that target animal hosts such as bovine viral diarrhea virus (BVDV), bluetongue virus (BTV), foot-and-mouth disease virus, porcine reproductive and respiratory syndrome virus (PRRSV), and porcine circovirus type 2 (PCV2) [144,145,146,147]. With continued improvements in testing technologies, powerful multiplex qPCR diagnostic techniques will help researchers to generate new knowledge regarding disease pathogenesis, and clinicians will be able to obtain more accurate and timely differential diagnoses for patients, ultimately resulting in more precise treatment plans and improved healthcare outcomes.

## Figures and Tables

**Table 1:** Herpesviridae oral manifestations. Summary of selected clinical cases that examined viral oral manifestations in humans with human herpesviruses.

Human Herpesviruses (Linear, Double-Stranded DNA, Enveloped Vírus)			
Herpes Simplex Virus-1 (HSV-1)			
Oral Manifestations	Signs and Symptoms	Disease Association	Treatment
Persistent mucocutaneous and oral lesions; recurrent herpetiform ulcerations (sold Sores)	Severe generalized pain in oral cavity, difficulty eating and opening mouth	Oral necrotizing disease	Valacyclovir (anti-viral); immunosuppressive and myeloablative agents (busulfan IV, fludarabine, fludarabine, keratinocyte growth factor), antibiotics (amoxicillin/clavulanate) (pre-transplant); superficial cleanings, chlorhexidine (post-transplant)
Infection of the gums	Tissue destruction, loss of connective tissues that support teeth	Aggressive periodontitis	Deep cleaning (scaling, root planing)
Oral nodular lesions; oral hairy leukoplakia	Recurrent oral ulcers, lip ulceration, painful nodular lesions on tongue, gums, and palate, fevers, rigors, inflammation	Immune Reconstitution inflammatory syndrome (IRIS)	High-dose cotrimoxazole and oral therapy, tenofovir/emtricitabine and efavirenz (antiretroviral), antituberculosis therapy, acyclovir
Neonatal gingival infection	Decreased eating/drinking, lethargy, vomiting, hypoxia in neonates; Fever, muscle and joint pain, lesions in oropharynx, along vermillion border	Primary herpetic gingivostomatitis (PHGS)	Valacyclovir for mother; acyclovir, ampicillin, gentamicin for neonate
Herpes Simplex Virus-2 (HSV-2)			
Oral Manifestations	Signs and Symptoms	Disease Association	Treatment
Painful infection of gingival and hard palate	Low fever, malaise, cervical lymphadenopathy, severely painful oral lesions, marginal alveolar bone loss	Acute herpetic gingivostomatitis (AHGS)/primary herpetic gingivostomatitis (PHGS)	Acyclovir (antiviral), betadine mouthwash, oral analgesics
Varicella-Zoster Virus (VSV)			
Oral Manifestations	Signs and Symptoms	Disease Association	Treatment
Infection of maxillary and mandibular branches of trigeminal nerve, oral vesicles	Mucosal vesicles and erosion, jaw osteonecrosis, teeth exfoliation, pulp calcification and necrosis, periapical lesions, lichen planus in oral cavity (gingival erythema and leukoplakia), severe pain	Herpes zoster infection (HZI); severe periodontitis; internal root resorption	Root canal
Epstein-Barr Virus (EBV)			
Oral Manifestations	Signs and Symptoms	Disease Association	Treatment
Periodontal infection	Persistent and severe inflammation of the gums due to immune response, alveolar bone resorption and loss of tooth attachment	Periodontitis (in Kostmann syndrome)/aggressive periodontitis	Scaling, deep cleaning, trimethoprim and sulfamethoxazole (antibiotics)
Endodontic infection	Endodontic tissue swelling	Apical periodontitis	Antibiotics, root canal
Endodontic lesions	Slow-healing oral lesions, endodontic abscesses	Pulp necrosis	Root canal, coronal restoration
Subgingival infection	Probing depths ≥ 6 mm, recurrent gum inflammation, no response to conventional treatment	Severe periodontitis	Valacyclovir (Valtrex^®^, antiviral)
Recurrent ulcers in oral mucosa	Sores due to ulcers in oral mucosa, lip and facial swelling	Lymphoproliferative disorder (can be mistaken for aggressive lymphoma)	Antibiotics (not successful)
Oral epithelial cell infection; lymphatic B cell infection	Sore throat, decreased appetite, fatigue, myalgia, cough, runny and stuffy nose	Infectious mononucleosis (kissing disease)	Corticosteroids (to open airways), valacyclovir
Oral mucosa infection	Oral mucosa lesion, soft and white, asymptomatic lesion, hairy surface usually on lateral aspect of tongue (lesions have been reported in oropharynx, soft palate, buccal mucosa, and mouth floor)	Oral hairy leukoplakia (OHL)	Valacyclovir, acyclovir, and desciclovir (antivirals)
Mediates nasopharyngeal carcinogenesis	Tumor growth, metastasis	Nasopharyngeal carcinoma (NPC)	Surgical removal (nasopharyngectomy), chemotherapy, immunotherapy (immune checkpoint inhibitor)
Human Cytomegalovirus (HCMV)			
Oral Manifestations	Signs and Symptoms	Disease Association	Treatment
Infection of the gums	Destruction of oral connective tissues, inflammation, tissue loss	Periodontitis (in Kostmann syndrome)	Scaling, deep cleaning, chlorhexidine rinse, trimethoprim and sulfamethoxazole (antibiotics)
Endodontic infection	Endodontic tissue swelling	Apical periodontitis	Root canal
Endodontic lesions	Slow-healing oral lesions, endodontic abscesses	Pulp necrosis	Root canal, coronal restoration
Oral ulcers following organ transplant	Oral lesions following renal transplant (lesions in buccal mucosa, hard and soft palate, tongue, and mouth floor)	Cytomegalovirus disease	Prednisone, Tacrolimus, Cyclosporin A, Azathioprine, Mycophenolate Mofetil (immunosuppressants), Ganciclovir (antiviral)
Human Herpesvirus-6 (HHV-6)			
Oral Manifestations	Signs and Symptoms	Disease Association	Treatment
Endodontic infection	Endodontic tissue swelling	Apical periodontitis	Root canal
Endodontic lesions	Slow-healing oral lesions, endodontic abscesses	Pulp necrosis	Root canal, coronal restoration
Human Herpesvirus-7 (HHV-7)			
Oral Manifestations	Signs and Symptoms	Disease Association	Treatment
Periodontium infection	Elevated viral expression as measured by PCR in gingival and periodontal tissue, tissue acting as a viral reservoir/infection site	None	None
Kaposi’s Sarcoma-Associated Herpesvirus (KSHV)			
Oral Manifestations	Signs and Symptoms	Disease Association	Treatment
Endodontic infection	Endodontic tissue swelling	Apical periodontitis	Root canal
Endodontic lesions	Slow-healing oral lesions, endodontic abscesses	Pulp necrosis	Root canal, coronal restoration

**Table 2:** SARS-CoV-2 oral manifestations. Summary of selected clinical cases that examined viral oral manifestations in humans with SARS-CoV-2.

SARS-CoV-2 (Linear, Single-Stranded RNA, Enveloped Vírus)			
Oral Manifestations	Signs and Symptoms	Disease Association	Treatment
Necrotizing gingivitis	Necrotic interdental papillae, bleeding in gingival sulcus and halitosis, erythematous and oedematous gingivae	Oral necrotizing disease	Debridement, deep cleaning (scaling, root planning), metronidazole or ampicillin, chlorhexidine mouthwash
Dark pigmentation	Dark brown pigmentation in the palate and gingiva	Oral melanotic macule, oral lichen planus, oral cancer	Ibuprofen
Xerostomia	Dry feeling in the mouth, frequent thirst, difficulty swallowing dry foods, diminished or altered taste	Periodontitis, caries, COVID-19	Artificial saliva substitutes
Dysgeusia	Altered taste perception (frequent metallic or bitter taste)	Periodontitis, COVID-19	Addressing cause of dysgeusia (e.g., vitamin supplements for vitamin deficiency, switching medications if dysgeusia is a side effect of medication)
Mucositis	Red pigmentation and swelling on affected mucous membranes, ulcers and sores	Gingivitis, periodontitis, COVID-19	Benzydamine, artificial saliva substitutes, low-level laser therapy
Periodontal infection	Persistent and severe inflammation of the gums due to immune response, alveolar bone resorption and loss of tooth attachment	Periodontitis (in COVID-19)	Scaling, deep cleaning, trimethoprim and sulfamethoxazole (antibiotics)
Erythematous bullae on palate	Erythema (redness), fluid-filled blisters, pain or tenderness on palate	Pemphigus vulgaris, HSV infection, COVID-19	Corticosteroids, acyclovir or valacyclovir, saline rinse
Tongue depapillation	Flat or glossy tongue surface, xerostomia, halitosis	Candida infection, COVID-19	Nystatin oral suspension or fluconazole

## Data Availability

No new data were created or analyzed in this study. Data sharing is not applicable to this article.
